# Aqueous Extract of Pomegranate Alone or in Combination with Citalopram Produces Antidepressant-Like Effects in an Animal Model of Menopause: Participation of Estrogen Receptors

**DOI:** 10.3390/ijms18122643

**Published:** 2017-12-19

**Authors:** Brenda Valdés-Sustaita, Carolina López-Rubalcava, María Eva González-Trujano, Cristina García-Viguera, Erika Estrada-Camarena

**Affiliations:** 1 Departamento de Farmacobiología, Centro de Investigación y Estudios Avanzados (CINVESTAV), Mexico City C.P.14330, Mexico; bvaldes@cinvestav.mx (B.V.-S.); clopezr@cinvestav.mx (C.L.-R.); 2 Laboratorio de Neurofarmacología de Productos Naturales, Dirección de Investigaciones en Neurociencias, Instituto Nacional de Psiquiatría “Ramón de la Fuente,” Mexico City C.P.14370, Mexico; evag@imp.edu.mx; 3 Department of Food Science and Technology, CEBAS-CSIC, 30100 Murcia, Spain; cgviguera@cebas.csic.es; 4 Laboratorio de Neuropsicofarmacología, Dirección de Investigaciones en Neurociencias, Instituto Nacional de Psiquiatría “Ramón de la Fuente,” Mexico City C.P.14370, Mexico

**Keywords:** pomegranate fruit, citalopram, ovariectomy, antidepressant-like action, estrogen receptors, polyphenols

## Abstract

It has been reported that the aqueous extract of pomegranate (AE-PG) has polyphenols with estrogenic-like activities. The present work determines if AE-PG alone or in combination with the selective serotonin reuptake inhibitor, citalopram, has antidepressant-like effects. It was also analyzed the participation of estrogen receptors (ER). AE-PG (0.1, 1.0, 10, or 100 mg/kg) was evaluated in ovariectomized female Wistar rats subjected to the forced swimming test. The effects induced by AE-PG were compared with those of citalopram (2.5, 5.0, 10, and 20.0 mg/kg) and 17β-estradiol (E2; 2.5 5.0, and 10 μg/rat). Likewise, the combination of suboptimal doses of AE-PG (0.1 mg/kg) plus citalopram (2.5 mg/kg) was evaluated. To determine if ER participates in the antidepressant-like action of pomegranate, the estrogen antagonist tamoxifen (15 mg/kg) was administered with AE-PG (1 mg/kg). AE-PG produced antidepressant-like actions with a similar behavioral profile induced by citalopram and E2. Suboptimal doses of citalopram plus AE-PG produced antidepressant-like effects. Tamoxifen was able to block AE-PG’s antidepressant-like actions. These results confirm the participation of ER in AE-PG’s antidepressant-like effects. Furthermore, the additive effects observed with the combined treatment of AE-PG plus citalopram could be advantageous in the treatment of depressive disorders, such as menopause.

## 1. Introduction

The pomegranate *Punica granatum* L. (*Lythraceae*) fruit possess several beneficial health effects for which it is considered a functional food [1]. Clinical and preclinical studies have shown that pomegranate has anti-oxidant [2], anti-inflammatory, anticarcinogenic, anti-microbial [1], anti-obesity [3], anti-nociceptive [4], and neuroprotective [5] actions. These properties are related to the high content of phytochemicals in all parts of the fruit; for example, the peel is rich in polyphenols like gallic acid, ellagic acid, catechin, epicatechin, quercetin, kaempferol, luteolin, rutin, and anthocyanins; some of them are a source of compounds with estrogenic activity. Additionally, the seeds are rich in fatty acids, sterols, and phytoestrogens, such as punicic acid, linoleic acid, oleic acid, palmitic acid, stigmasterol, β-sitosterol, cholesterol, 17-α-estradiol, estrone, testosterone, estriol, oleanolic acid, isoflavones, and phenyl aliphatic glycosides/lignins [3,6,7,8,9].

Interestingly, the main polyphenols present in the aqueous extract of the fruit of pomegranate are ellagitannins, such as punicalagin (α and β), and flavonoids, such as anthocyanidins, catechins, and flavonols [4]. Ellagitannins are easily hydrolyzed to ellagic acid and in different portions of the small and large intestine; ellagitannins are transformed by bacterial metabolism to hydroxy-6H-dibenzo[*b.d*]pyrene-6-one compounds, called urolithins. These last compounds possess estrogenic activity on in vitro assays [10,11]. These data suggest that pomegranate is an excellent source of phytoestrogens [12,13].

On the other hand, the first-line treatments prescribed against menopause symptoms are antidepressants, such as selective serotonin reuptake inhibitors to treat depressive symptoms and hormone replacement therapy for hot flashes and osteoporosis [14,15]. However, in the last decades, the use of phytoestrogens recovers new interest due to its potential for the treatment of osteoporosis and vasomotor symptoms among others [16]. In fact, the therapeutic effect of pomegranate for the treatment of menopause-related symptoms has been tested with controversial results. For example, a systematic review reported that pomegranate treatment reduces osteoporosis, osteoarthritis, or rheumatoid arthritis [17]; in contrast, pomegranate treatment was ineffective to reduced hot flashes and psychiatric symptoms [18].

In parallel, preclinical studies have reported that pomegranate reduced some menopausal signs in animal models. For instance, pomegranate seed oil consumption favors bone mineral density and prevents the trabecular damage in ovariectomized mice [19]. Additionally, the juice of pomegranate, rich in phytoestrogens, produces antidepressant-like effects and decreases bone loss in ovariectomized mice [8]. In both studies, the pomegranate was administered immediately after surgery; however, it is unknown if the pomegranate juice can induce antidepressant-like effects when its administration starts several weeks after ovary elimination. Furthermore, although phytoestrogens have been proposed to mediate the antidepressant-like action of pomegranate, the involvement of estrogen receptors in its mechanism of action is unknown.

Nevertheless, Naveen et al. reported that the juice of pomegranate-peel extract induced antidepressant-like actions in the chronic mild stress model [20]. These authors suggested that this antidepressant-like effect was due to the high content of polyphenols, mainly flavonoids which are proposed to decrease the monoamine oxidase (MAO)-A and -B activity in the brain, modifying the monoamine levels [20]. These results suggest that as a modulator of monoamine levels, pomegranate administration could synergize with antidepressant drugs to facilitate an antidepressant-like effect.

Therefore, the present work evaluates if the aqueous extract of *Punica granatum* (AE-PG) alone or in combination with the selective serotonin reuptake inhibitor, citalopram, produces an antidepressant-like effect and if this effect is related to estrogen receptor (ER) activation in ovariectomized rats. As reference drugs, citalopram and 17β-estradiol (E2), were tested on the forced swimming test (FST) according to previous reports [21,22,23].

## 2. Results

### 2.1. Antidepressant-Like Effect of the AE-PG, E2, and Citalopram

**Figure 1:**
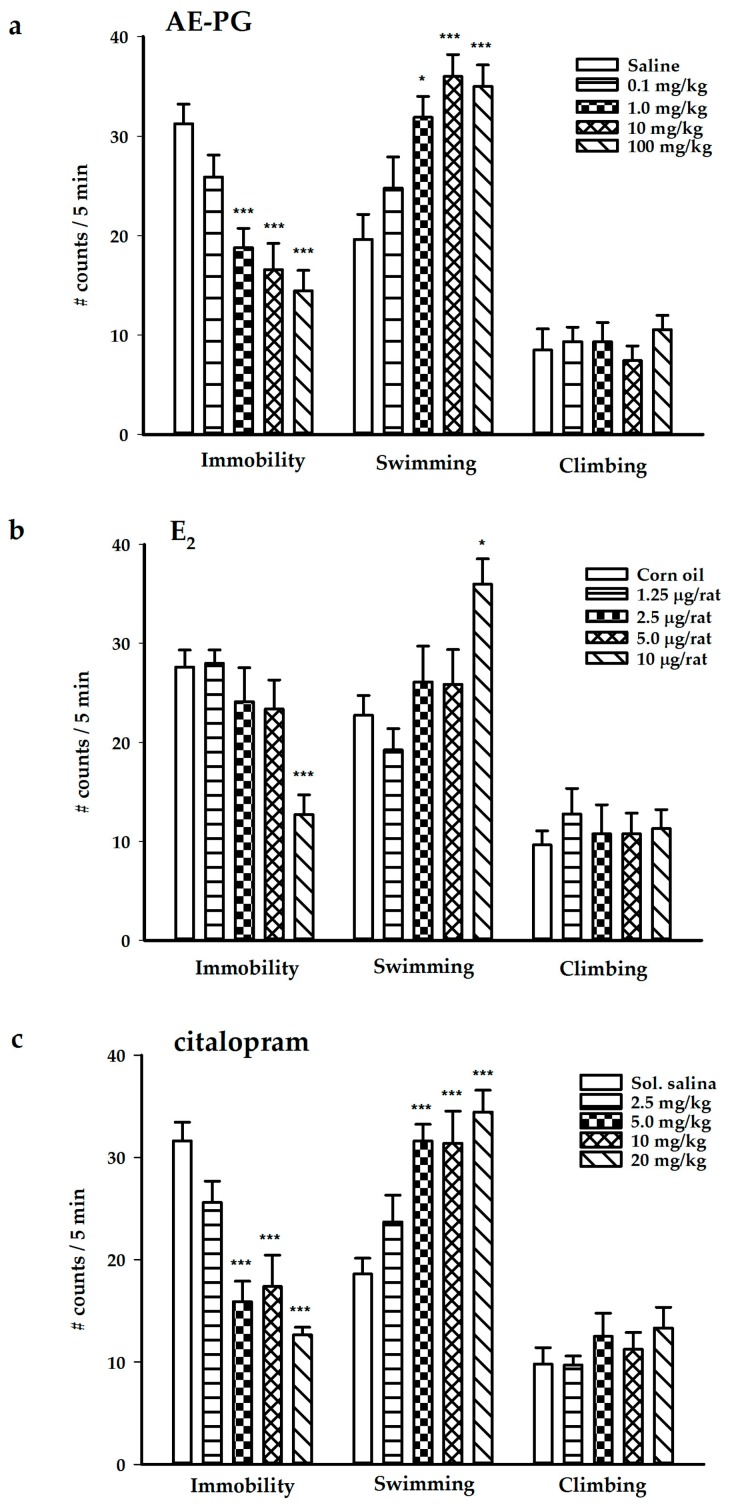
Behavioral effects produced by the chronic administration (14 days; one administration per day) of the AE-PG (panel (**a**); *n* = 7–9 per dose), E2 (panel (**b**); *n* = 7–8 per dose), and citalopram (panel (**c**); *n* = 8–10 per dose) on the forced swimming test. The figure shows the mean number of counts ± standard error. Dunnett post hoc: * *p* < 0.05; *** *p* < 0.001 versus the control group. AE-PG = aqueous extract of pomegranate; E2 = 17β-estradiol.

The chronic administration of the AE-PG at the doses of 1, 10, and 100 mg/kg produced a significant decrease in immobility (antidepressant-like effect) (F_(4,37)_ = 10.366; *p* < 0.001). In addition, the AE-PG produced an increase in swimming behavior (F_(4,37)_ = 7.705; *p* < 0.001) without modifying the climbing behavior (F_(4,37)_ = 0.428; non-significant) (Figure 1a). In contrast, the chronic administration of E2 at the dose of 10 μg/rat produced an antidepressant-like effect (decreased immobility: F_(4,34)_ = 6.039; *p* < 0.001) increasing the swimming behavior (F_(4,34)_ = 4.478; *p* < 0.05), but not the climbing behavior (F_(4,34)_ = 0.253; non-significant) (Figure 1b). Finally, the chronic administration of citalopram also produced an antidepressant-like effect at the doses of 5, 10, and 20 mg/kg by decreasing immobility (F_(4,42)_ = 15.131; *p* < 0.001) and increasing swimming behavior (F_(4,42)_ = 8.967; *p* < 0.001) while the climbing behavior was not modified by any dose (F_(4,42)_ = 0.868; non-significant) (Figure 1c).

### 2.2. Participation of ER in the Antidepressant-Like Effect of AE-PG

**Figure 2:**
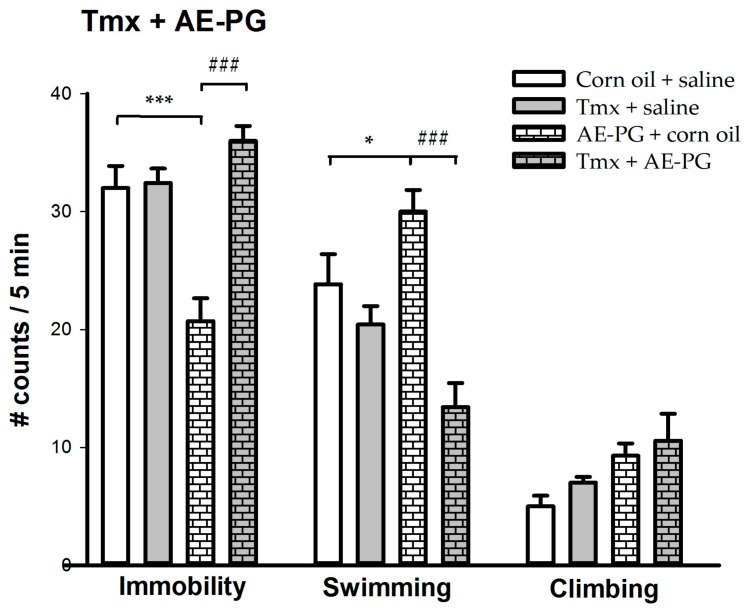
Behavioral effects of the chronic administration (14 days; one administration per day) of the minimum effective dose of the AE-PG (1.0 mg/kg) with Tmx (15 mg/kg) on the forced swimming test. The figure shows the number of counts ± standard error (*n* = 6–7 animals per dose). Tukey post hoc: * *p* < 0.05; *** *p* < 0.001 versus the corn oil + saline group; ^###^ *p* < 0.001 versus the AE-PG + corn oil group. AE-PG = aqueous extract of pomegranate; Tmx = Tamoxifen.

Figure 2 shows the effect of the chronic administration of the minimum effective dose of AE-PG (1 mg/kg) in the presence of a non-selective ER antagonist (tamoxifen; 15 mg/kg). The group treated only with the AE-PG decreased in immobility when compared to the vehicles groups, by contrast, the group treated with tamoxifen shows a similar behavioral pattern to the control group. Importantly, the group treated with AE-PG in the presence of tamoxifen blocked the antidepressant-like effect produced by the group treated with AE-PG alone. The one-way ANOVA shows significant differences in immobility (F_(3,23)_ = 17.841; *p* < 0.001) and in swimming behavior (F_(3,23)_ = 12.470; *p* < 0.001), but not in climbing behavior (F_(3,23)_ = 3.022; non-significant).

### 2.3. Effect of the Simultaneous Administration of Suboptimal Doses of AE-PG plus Citalopram

**Figure 3:**
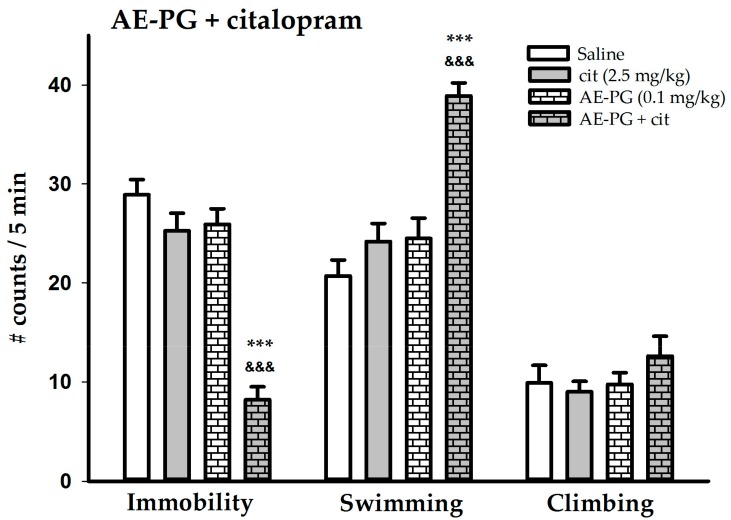
Behavioral effects of the simultaneous administration of the suboptimal dose of the AE-PG (0.1 mg/kg) plus the suboptimal dose of the citalopram (2.5 mg/kg) (chronic administration for 14 days; one administration per day) on the forced swimming test. Shown are the mean number of counts ± standard error (*n* = 10 animals per dose). Tukey post hoc: *** *p* < 0.001 versus the control group; ^&&&^ versus the suboptimal doses groups. AE-PG = aqueous extract of pomegranate.

Figure 3 shows that the chronic administration of the AE-PG (0.1 mg/kg) and citalopram (2.5 mg/kg) given independently did not produce any change in any behavior. The ANOVA results are: F_(3,36)_ = 39.908, *p* < 0.001 for immobility; and F_(3,36)_ = 20.217, *p* < 0.001 for swimming and F_(3,36)_ = 0.907, NS for climbing. The group treated with combination of the suboptimal doses of the AE-PG and the citalopram produced additive effects since there was a significant decrease in immobility (Tukey’s test: *p* < 0.001; citalopram + AE-PG vs. AE-PG + saline) (*p* < 0.001; citalopram + AE-PG vs. AE-PG + saline) and the swimming behavior increased (Tukey’s test: *p* < 0.001; citalopram + AE-PG vs. AE-PG + saline) (*p* < 0.001; citalopram + AE-PG vs. AE-PG + saline) when compared to the independent groups.

### 2.4. Effects of Treatments on the General Activity Test

No significant differences in locomotor activity were shown between the control group and the treated groups with the AE-PG and with E2 (Table 1). However, the chronic administration of citalopram at the dose of 5 mg/kg produced a significant decrease in locomotor activity (Table 1). On the other hand, neither the chronic administration with AE-PG plus citalopram nor with AE-PG plus citalopram produced significant differences in locomotor activity when compared to control groups (Table 2).

## 3. Discussion

The present study determined if the aqueous extract of pomegranate (AE-PG) induced antidepressant-like effects in rats under conditions similar to those of menopause in women. To this aim, we used the forced swimming test to analyze the effects of AE-PG. This animal model of depression is one of the most common models used for evaluating pharmacological antidepressant activity [24,25,26] and it provides a tool to analyze the participation of different neurochemical circuits involved in their mechanism of action [27,28]. Thus, it has been established that swimming behavior is increased by a serotonergic mechanism, while climbing behavior is increased by a catecholaminergic one [22,24,29,30].

The results of the present study confirm that the AE-PG induces antidepressant-like effects and that ER participate in this effect probably through an interaction with the serotonergic system. Moreover, it was determined that the combination of AE-PG plus citalopram produce an additive antidepressant-like effect.

### 3.1. Antidepressant-Like Action Induced by Chronic Treatment of AE-PG

The chronic administration of the aqueous extract of pomegranate reduced immobility behavior and significantly increased swimming behavior without modifying the climbing behavior. A similar effect was induced by the typical serotonergic antidepressant citalopram. This result suggests that the pomegranate extract produces an antidepressant-like effect in ovariectomized rats. These results are consistent with two studies in male and female mice that showed an antidepressant-like effect produced by pomegranate extract under an acute treatment [8]. However, that study did not analyze the active behaviors of the aqueous extract in the forced swimming test.

Based on the behavioral profile observed in the present study, it can be suggested that AE-PG induces its antidepressant-like actions through the serotonergic system. Additionally, the behavioral effects observed were similar to those induced by the selective serotonin reuptake inhibitor citalopram and by E2. In a previous study the participation of the serotonergic system in E2 antidepressant-like effect was demonstrated [22,31]. Therefore, the idea that the serotonergic system could participate in the antidepressant-like effect of AE-PG is feasible.

Currently, there are no reports in the literature showing that pomegranate extract has a direct action on the serotonergic system. A study in male mice evaluated the effects of the polyphenols obtained from the peel of the pomegranate. In this case, it was shown that pomegranate extract induces an antidepressant-like effect by inhibiting brain MAO-B and MAO-A enzyme activity by 60% and 17%, respectively [20]. It is possible that the inhibition of both enzymes’ activity contributes to prevent 5-HT degradation and, therefore, an increase in swimming behavior could be observed, however, specific experiments are required to support this proposal.

In this study, we used an aqueous extract of the whole pomegranate. According to González-Trujano et al. the polyphenols that predominate in this extract are ellagitannins (ellagic acid in its two forms: glycone and aglycone; 15.77 ± 0.15 mg/g) and punicallagins (5.46 ± 0.04 mg/g); and to a lesser extent, the flavonoids anthocyanidins (2.09 ± 0.23 mg/g) [4]. These data agree with another report in which the extract of the whole fruit of pomegranate contains flavonoids and ellagitannins as total soluble polyphenols [32].

Ellagic acid has been tested in the FST in mice and it has been seen that it induces an antidepressant-like effect prevented by the serotonin depletion and the antagonism to 5-HT2, 5-HT3, and α1- and α2-adrenoceptors [33]. This data suggests that this photochemistry could be responsible, at least in part, of the antidepressant-like action of AE-PG.

Another possible explanation for the effect of AE-PG on swimming behavior could be given if one considers that ellagitannins can be metabolized by the microbiota to compounds called urolithins, which have activity on estrogen receptors α and β [11,34]. Indeed, ERβ agonists have similar behavioral effects as AE-PG in the FST [35]. Additionally, the phytoestrogens genistein and coumestrol have a similar effect in the FST [35,36,37].

There is evidence that ERβ activation promotes changes in the 5-HT-1A receptor’s sensitivity [38], a receptor involved in the antidepressant-like effect of E2 [31]. Considering this information, it is possible that the metabolites of the ellagitannins induce their antidepressant-like effect through the activation of estrogen receptors, which modulate the serotonergic system activity [39]. In fact, our results reveal that the antidepressant-like effect produced by the AE-PG is reversed with the administration of tamoxifen, a non-specific estrogen receptor antagonist [40], confirming the participation of ER in this effect.

Furthermore, it should be mentioned that current results with the chronic administration of citalopram contrast with the results previously reported [41]. Vega-Rivera et al. showed that citalopram had no action in the FST: however, in that study 15 month-old female rats and a sub-chronic scheme of drug treatment was used. In contrast, in the present study we used young female rats and administered a chronic treatment. Therefore, it is likely that both the age and the treatment regimen could modify citalopram’s response. Future studies should analyze this proposal.

Our results are consistent with previous studies following sub-chronic citalopram administration, even at lower doses than those handled in the present study [42,43].

### 3.2. Combined Treatment of AE-PG plus Citalopram

The combination of suboptimal doses of AE-PG and citalopram induced antidepressant-like effects; these actions were significantly higher in comparison with the individual treatments of E2 and citalopram per se. In this case, the combined treatment decreased immobility behavior by 31.9% compared to AE-PG and by 31.66% compared to citalopram. Therefore, we could say that it was observed an additive antidepressant-like effect. Concerning swimming behavior, the combination produced an increase of 62.1% compared to the AE-PG and 65.5% compared to citalopram.

Generally, in clinical practice, the first choice of drug for the treatment of depressive disorders during menopause is citalopram, because its metabolism is mainly through the cytochrome p450 isoenzyme CYP2C19. Therefore, citalopram is the antidepressant that least intervenes in the metabolism of drugs prescribed for other medical complications common during menopause, such as cardiovascular illnesses [44].

With regard to the metabolism of polyphenols, they are known to be metabolized principally through the intestinal microbiota [45]; the main route for the ellagitannins [46]. It is important to mention that it has been determined that polyphenols inhibit the activity of the CYP3A4 enzyme [47]. In particular, the ellagic acid is known to have an inhibitory potential on the CYP1A1 and CYP2E1 enzymes [48].

An advantage of this combination is that due to the behavioral response observed and based on the literature, the inhibitory effect of the polyphenols contained in the AE-PG does not interfere with the metabolism of citalopram, so their combined use could be safe. In clinical and preclinical studies, it has been established that the combination of selective serotonin reuptake inhibitors and estrogenic compounds produce a potentiation of the antidepressant effect [49,50]. In particular, Soares et al. determined that citalopram could be effective as an adjunctive treatment for vasomotor symptoms and mood disorders in women during peri- and post-menopause who did not respond to the treatment with E2 alone [51].

Taking into account that, in the present study, AE-PG produces its antidepressant effect through the serotoninergic system and, with the participation of ER, our results showed a pattern similar to the results obtained by Recamier-Carballo et al. In this case, an additive action of the antidepressant-like effects of a selective serotonin reuptake inhibitor (fluoxetine) occurred with E2 [49]. Thus, the combination AE-PG plus citalopram could be an alternative that involves the use of a natural product with estrogenic activity.

Finally, regarding the results of animals’ locomotion, in the present study, it was observed that citalopram decreased locomotion but, at the same time, in the FST, it decreases immobility by increasing swimming behavior. In this case, the effect of citalopram in locomotion is not affecting its antidepressant-like effect because a sedative effect would be seen in the FST by an increase in immobility behavior and decreased active behaviors [24]. Therefore, it is possible to rule out that the antidepressant-like effect of this compound is due to a non-specific action.

## 4. Materials and Methods

### 4.1. Animals

This study used ovariectomized female Wistar rats (12–16 weeks old). All animals were provided by the vivarium of the “Instituto Nacional de Psiquiatría Ramón de la Fuente Muníz (INPRFM)” (Mexico City, Mexico) and housed in groups of eight in polycarbonate cages. Animals were maintained on a 12 h inverted light-dark cycle (lights on at 10 am) in a 23–25 °C temperature-controlled room of the “Centro de Investigación y Estudios Avanzados del Instituto Politécnico Nacional (CINVESTAV-IPN)” (Mexico City, Mexico). Rats had free access to food and water. All animal procedures were performed according to the official Mexican norm (NOM-062-ZOO-1999) and approved by the Ethics Committee of the CINVESTAV-IPN No. 379-07 (23/04/2007) and INPRFM No. CEI-200 (10/12/2015).

### 4.2. Drugs

In this study we used a whole fruit aqueous extract of *Punica granatum* (AE-PG) which was kindly provided by Nutracitrus S.L. (Elche, Alicante, Spain). The AE-PG was dissolved in saline solution 0.9% and administered orally (0.1, 1, 10, and 100 mg/kg doses); 17-β estradiol or E2 (Sigma-Aldrich, Toluca, Mexico) was dissolved in corn oil and injected subcutaneously (1.25, 2.5, 5, and 10 μg/rat). The citalopram hydrobromide (Sigma-Aldrich, Toluca, Mexico) was dissolved in saline solution 0.9% and injected intraperitoneally (2.5, 5, 10, and 20 mg/kg), and tamoxifen citrate (Sigma-Aldrich, Toluca, Mexico) was dissolved in corn oil and injected subcutaneously (15 mg/kg dose). The anesthetic 2,2,2-Tribrom-ethanol 2% (Sigma-Aldrich, Toluca, Mexico) was freshly prepared (for 25 mL, 0.5 g of tribrom-ethanol were dissolved in 2 mL of ethanol; thereafter, 23 mL of saline solution was slowly added while stirring) and administered intraperitoneally (10 mL/kg).

### 4.3. Ovariectomy

Ovariectomy was performed under 2,2,2-Tribromo-ethanol 2% (200 mg/kg) anesthesia. Ovaries were removed from the low abdominal cavity through one single midline incision which was properly sutured and treated with benzalkonium chloride 50%. Animals were kept in recovery for three weeks and then randomly divided into the experimental groups, and the behavioral studies were performed.

### 4.4. Forced Swimming Test (FST)

The animals were individually placed in a glass-cylinder (45 cm height × 20 cm diameter) with water at 25 ± 1 °C and a depth of 30 cm. The FST was held in two sessions; the pre-test session, which lasted for 15 min and the test session, which lasted 5 min and which was performed fourteen days after the pre-test. There are several report that indicate that the changes induced by the pre-test remains for at least 21 days [52,53,54], to revert these changes the treatment started after the pre-test session. The test session was video-recorded for later analysis. After each session, the rats were removed from the water, well dried with paper towels, and returned to their clean home cages.

### 4.5. Behavioral Scoring

The behavioral scoring was performed to the test session by a five second sampling technique in which immobility, swimming, and climbing behaviors were recorded by an observer that was unaware of the groups’ treatments. Immobility behavior was considered when the rat did just the necessary movements to stay afloat, swimming behavior was considered when the rat moved around the cylinder or dived, and climbing behavior was considered when presenting active movements of scaling, such as moving in and out of the water with its forepaws against the glass walls [55].

### 4.6. Activity Test

The effect of drug treatments on locomotor activity was evaluated in the open-field test just before the test session of the FST and video-recorded for later analysis. Briefly, animals were individually placed for 5 min in an acrylic cage (43 × 33 × 20 cm) with a drawn grid on the floor with 12 squares (11 × 11 cm). For the behavioral analysis, the number of times that the rat crossed the drawn squares was recorded during the 5 min session. A count was considered when the four paws were in each square. Between each session, the cage was cleaned with cleaning solution.

### 4.7. Statistics

Statistical analysis was performed using the Sigma Plot 12.0 Systat software (San Jose, CA, USA). One-way ANOVA followed by a Dunnet post hoc test was used when comparing experimental groups versus the control group, and one-way ANOVA followed by a Tukey’s post hoc test when making multiple comparisons between treatments. Values of *p* < 0.05 were considered as significant.

### 4.8. Experimental Design

#### 4.8.1. Experiment 1: Antidepressant-Like Effects of AE-PG, E2, and Citalopram

For this experiment, dose-response curves were constructed as follows: (1) AE-PG (saline, 0.1, 1, 10, and 100 mg/kg); (2) E2 (corn oil, 1.25, 2.5, 5, and 10 μg/rat) as a positive control of antidepressant-like effect of estrogenic compounds; and (3) citalopram (saline, 2.5, 5, 10, and 20 mg/kg) as a positive control of antidepressant-like effects of selective serotonin reuptake inhibitors. All treatments were conducted with an independent group design (*n* = 7–10 rats per dose) and followed a chronic administration regimen (one dose per day during 14 days) that started twenty-four hours after the pre-test session. All behavioral tests were conducted twenty-four hours after the last pharmacological administration.

#### 4.8.2. Experiment 2: Analysis of the Participation of ER in the Antidepressant-Like Effects of AE-PG

Once the antidepressant-like effect of AE-PG was determined, the minimum effective dose of AE-PG (1 mg/kg) was evaluated in the presence of the non-selective ER antagonist; tamoxifen (tamoxifen; 15 mg/kg). For this experiment, we used four independent experimental groups of ovariectomized Wistar rats: (1) saline + corn oil; (2) tamoxifen + saline; (3) AE-PG + corn oil, and (4) tamoxifen + AE-PG. All experimental groups (*n* = 7–10 animals per group) followed the same experimental design of the chronic scheme of treatment used in Experiment 1.

#### 4.8.3. Experiment 3: Effect of the Simultaneous Administration of Suboptimal Doses of AE-PG Plus Citalopram

We evaluated the antidepressant-like effect of the combination of the suboptimal doses of AE-PG (0.1 mg/kg) and citalopram (2.5 mg/kg) determined from the dose-response curves constructed in Experiment 1. Additionally, four experimental groups of ovariectomized Wistar rats were used: (1) Saline; (2) AE-PG + saline; (3) citalopram + saline and (4) AE-PG + citalopram. All experimental groups (*n* = 10–12 animals per group) followed the same experimental design of the chronic scheme of treatment used in Experiment 1.

## 5. Conclusions

This study determined that the chronic administration of AE-PG produces an antidepressant-like effect through the modulation of the serotoninergic system and the participation of the ER. The results suggest that metabolites derived from the metabolism of ellagitannins (urolithins) may have estrogenic action. However, further studies are needed to confirm their direct involvement.

Likewise, an antidepressant-like effect was observed when the pomegranate extract was combined with citalopram, suggesting an additive-type action, since we observed a summation of about 50%. According to the literature, when combining the compounds, the metabolism of one compound does not alter the metabolism of the other. However, it would be necessary to confirm that they do not have undesirable pharmacokinetic interactions.

## Figures and Tables

**Table 1:** Number of crossings in the open-field test with the chronic administration of AE-PG, 17β-estradiol, and citalopram.

Treatment	Dose	*n*	Mean ± SEM	One Way ANOVA (F)	*p*
AE-PG	Saline	8	54.667 ± 5.632	F_(4,37)_ = 0.869	0.491
	0.1 mg/kg	9	53.667 ± 5.370		
	1 mg/kg	9	59.778 ± 7.579		
	10 mg/kg	7	60.556 ± 3.775		
	100 mg/kg	9	67.333 ± 6.298		
17β-estradiol	Corn oil	8	79.375 ± 1.711	F_(4,34)_ = 0.628	0.646
	1.25 μg/rat	8	77.750 ± 3.299		
	2.5 μg/rat	8	74.375 ± 3.615		
	5 μg/rat	8	75.125 ± 2.702		
	10 μg/rat	7	79.714 ± 3.656		
Citalopram	Saline	10	77.500 ± 3.390	F_(4,42)_ = 8.197	-
	2.5 mg/kg	10	82.400 ± 2.864		0.618
	5 mg/kg	10	60.500 ± 2.051		<0.05
	10 mg/kg	8	70.625 ± 2.179		0.376
	20 mg/kg	9	79.333 ± 4.444		0.982

The mean ± standard error, F value of the one-way ANOVA and Dunnett’s post hoc test vs. the control group are shown.

**Table 2:** Number of crossings in the open-field test with the chronic administration of Tmx + AE-PG and citalopram + AE-PG.

Experiment	Treatment	*n*	Mean ± SEM	One Way ANOVA (F)	*p*
Tmx + AE-PG	Corn oil + Saline	6	74.000 ± 2.944	F_(3,23)_ = 0.644	0.594
	Corn oil + AE-PG	7	75.000 ± 0.093		
	Tmx + Saline	7	78.143 ± 1.438		
	Tmx + AE-PG	7	74.429 ± 2.608		
AE-PG + Cit	Saline	10	62.200 ± 6.277	F_(3,36)_ = 4.797	0.07
	Saline + AE-PG	10	57.400 ± 6.848		
	Saline + citalopram	10	79.700 ± 2.745		
	AE-PG + citalopram	10	78.200 ± 3.408		

The mean ± standard error, F value of the one-way ANOVA and Dunnett’s post hoc test vs. the control group are shown.

## References

[1] Viladomiu M., Hontecillas R., Lu P., Bassaganya-Riera J. (2013). Preventive and prophylactic mechanisms of action of pomegranate bioactive constituents. Evid. Based Complement. Alternat. Med..

[2] Zhang L.H., Fu Q.J., Zhang Y.H. (2011). Composition of anthocyanins in pomegranate flowers and their antioxidant activity. Food Chem..

[3] Al-Muammar M.N., Khan F. (2012). Obesity: The preventive role of the pomegranate (*Punica granatum*). Nutrition.

[4] Gonzalez-Trujano M.E., Pellicer F., Mena P., Moreno D.A., Garcia-Viguera C. (2015). Antinociceptive and anti-inflammatory activities of a pomegranate (*Punica granatum* L.) extract rich in ellagitannins. Int. J. Food Sci. Nutr..

[5] Yuan T., Ma H., Liu W., Niesen D.B., Shah N., Crews R., Rose K.N., Vattem D.A., Seeram N.P. (2016). Pomegranate’s Neuroprotective Effects against Alzheimer’s Disease Are Mediated by Urolithins, Its Ellagitannin-Gut Microbial Derived Metabolites. ACS Chem. Neurosci..

[6] Viuda-Martos M., Fernandez-Lopez J., Perez-Alvarez J.A. (2010). Pomegranate and its Many Functional Components as Related to Human Health: A Review. Compr. Rev. Food Sci. Food Saf..

[7] Choi D.W., Kim J.Y., Choi S.H., Jung H.S., Kim H.J., Cho S.Y., Kang C.S., Chang S.Y. (2006). Identification of steroid hormones in pomegranate (*Punica granatum*) using HPLC and GC-mass spectrometry. Food Chem..

[8] Mori-Okamoto J., Otawara-Hamamoto Y., Yamato H., Yoshimura H. (2004). Pomegranate extract improves a depressive state and bone properties in menopausal syndrome model ovariectomized mice. J. Ethnopharmacol..

[9] Van Elswijk D.A., Schobel U.P., Lansky E.P., Irth H., van der Greef J. (2004). Rapid dereplication of estrogenic compounds in pomegranate (*Punica granatum*) using on-line biochemical detection coupled to mass spectrometry. Phytochemistry.

[10] Landete J.M. (2011). Ellagitannins, ellagic acid and their derived metabolites: A review about source, metabolism, functions and health. Food Res. Int..

[11] Larrosa M., Gonzalez-Sarrias A., Garcia-Conesa M.T., Tomas-Barberan F.A., Espin J.C. (2006). Urolithins, ellagic acid-derived metabolites produced by human colonic microflora, exhibit estrogenic and antiestrogenic activities. J. Agric. Food Chem..

[12] Espin J.C., Larrosa M., Garcia-Conesa M.T., Tomas-Barberan F. (2013). Biological significance of urolithins, the gut microbial ellagic acid-derived metabolites: The evidence so far. Evid. Based Complement. Alternat. Med..

[13] Heftmann E., Ko S.T., Bennett R.D. (1966). Identification of Estrone in Pomegranate Seeds. Phytochemistry.

[14] Handley A.P., Williams M. (2015). The efficacy and tolerability of SSRI/SNRIs in the treatment of vasomotor symptoms in menopausal women: A systematic review. J. Am. Assoc. Nurse Pract..

[15] Roberts H., Hickey M. (2016). Managing the menopause: An update. Maturitas.

[16] Lethaby A., Marjoribanks J., Kronenberg F., Roberts H., Eden J., Brown J. (2013). Phytoestrogens for menopausal vasomotor symptoms. Cochrane Database Syst. Rev..

[17] Shuid A.N., Mohamed I.N. (2013). Pomegranate Use to Attenuate Bone Loss in Major Musculoskeletal Diseases: An Evidence-Based Review. Curr. Drug Targets.

[18] Auerbach L., Rakus J., Bauer C., Gerner C., Ullmann R., Wimmer H., Huber J. (2012). Pomegranate seed oil in women with menopausal symptoms: A prospective randomized, placebo-controlled, double-blinded trial. Menopause.

[19] Spilmont M., Leotoing L., Davicco M.J., Lebecque P., Mercier S., Miot-Noirault E., Pilet P., Rios L., Wittrant Y., Coxam V. (2013). Pomegranate seed oil prevents bone loss in a mice model of osteoporosis, through osteoblastic stimulation, osteoclastic inhibition and decreased inflammatory status. J. Nutr. Biochem..

[20] Naveen S., Siddalingaswamy M., Singsit D., Khanum F. (2013). Anti-depressive effect of polyphenols and omega-3 fatty acid from pomegranate peel and flax seed in mice exposed to chronic mild stress. Psychiatry Clin. Neurosci..

[21] Ramirez-Rodriguez G., Vega-Rivera N.M., Oikawa-Sala J., Gomez-Sanchez A., Ortiz-Lopez L., Estrada-Camarena E. (2014). Melatonin synergizes with citalopram to induce antidepressant-like behavior and to promote hippocampal neurogenesis in adult mice. J. Pineal Res..

[22] Estrada-Camarena E., Fernandez-Guasti A., Lopez-Rubalcava C. (2003). Antidepressant-like effect of different estrogenic compounds in the forced swimming test. Neuropsychopharmacology.

[23] Estrada-Camarena E., Fernandez-Guasti A., Lopez-Rubalcava C. (2004). Interaction between estrogens and antidepressants in the forced swimming test in rats. Psychopharmacology.

[24] Cryan J.F., Valentino R.J., Lucki I. (2005). Assessing substrates underlying the behavioral effects of antidepressants using the modified rat forced swimming test. Neurosci. Biobehav. Rev..

[25] Rygula R., Abumaria N., Flugge G., Fuchs E., Ruther E., Havemann-Reinecke U. (2005). Anhedonia and motivational deficits in rats: Impact of chronic social stress. Behav. Brain Res..

[26] Bogdanova O.V., Kanekar S., D’Anci K.E., Renshaw P.F. (2013). Factors influencing behavior in the forced swim test. Physiol. Behav..

[27] Reneric J.P., Lucki I. (1998). Antidepressant behavioral effects by dual inhibition of monoamine reuptake in the rat forced swimming test. Psychopharmacology.

[28] Detke M.J., Lucki I. (1996). Detection of serotonergic and noradrenergic antidepressants in the rat forced swimming test: The effects of water depth. Behav. Brain Res..

[29] Chaki S., Yoshikawa R., Hirota S., Shimazaki T., Maeda M., Kawashima N., Yoshimizu T., Yasuhara A., Sakagami K., Okuyama S. (2004). MGS0039: A potent and selective group II metabotropic glutamate receptor antagonist with antidepressant-like activity. Neuropharmacology.

[30] Reneric J.P., Bouvard M., Stinus L. (2002). In the rat forced swimming test, NA-system mediated interactions may prevent the 5-HT properties of some subacute antidepressant treatments being expressed. Eur. Neuropsychopharm..

[31] Estrada-Camarena E., Fernandez-Guasti A., Lopez-Rubalcava C. (2006). Participation of the 5-HT1A receptor in the antidepressant-like effect of estrogens in the forced swimming test. Neuropsychopharmacology.

[32] Mondragon-Jacobo C., Hernandez-Herrera G., Guzman-Maldonado S.H. (2015). Agronomical, Physicochemical, and Functional Characterization of Mexican Pomegranates as Compared to Wonderful Pomegranate. Acta Hortic..

[33] Girish C., Raj V., Arya J., Balakrishnan S. (2012). Evidence for the involvement of the monoaminergic system, but not the opioid system in the antidepressant-like activity of ellagic acid in mice. Eur. J. Pharmacol..

[34] Dellafiora L., Mena P., Cozzini P., Brighenti F., del Rio D. (2013). Modelling the possible bioactivity of ellagitannin-derived metabolites. In silico tools to evaluate their potential xenoestrogenic behavior. Food Funct..

[35] Walf A.A., Rhodes M.E., Frye C.A. (2004). Antidepressant effects of ERβ-selective estrogen receptor modulators in the forced swim test. Pharmacol. Biochem. Behav..

[36] Wu Z.M., Ni G.L., Shao A.M., Cui R. (2017). Genistein alleviates anxiety-like behaviors in post-traumatic stress disorder model through enhancing serotonergic transmission in the amygdala. Psychiatry Res..

[37] Hu P., Ma L., Wang Y.G., Ye F., Wang C., Zhou W.H., Zhao X. (2017). Genistein, a dietary soy isoflavone, exerts antidepressant-like effects in mice: Involvement of serotonergic system. Neurochem. Int..

[38] Creech R.D., Li Q., Carrasco G.A., Van de Kar L.D., Muma N.A. (2012). Estradiol induces partial desensitization of serotonin 1A receptor signaling in the paraventricular nucleus of the hypothalamus and alters expression and interaction of RGSZ1 and G alpha z. Neuropharmacology.

[39] López-Rubalcava C., Vega-Rivera N.M., Páez-Martínez N., Estrada-Camarena E., Ru-Bans L. (2012). Participation of the Monoaminergic System in the Antidepressant-Like Actions of Estrogens: A Review in Preclinical Studies. Effects of Antidepressants.

[40] Lemini C., Cruz-Lopez B., Martinez-Mota L. (2013). Participation of estrogen receptors in the antidepressant-like effect of prolame on the forced swimming test. Pharmacol. Biochem. Behav..

[41] Vega Rivera N.M., Tenorio A.G., Fernandez-Guasti A., Estrada-Camarena E. (2016). The Post-Ovariectomy Interval Affects the Antidepressant-Like Action of Citalopram Combined with Ethynyl-Estradiol in the Forced Swim Test in Middle Aged Rats. Pharmaceuticals.

[42] Flores-Serrano A.G., Vila-Luna M.L., Alvarez-Cervera F.J., Heredia-Lopez F.J., Gongora-Alfaro J.L., Pineda J.C. (2013). Clinical doses of citalopram or reboxetine differentially modulate passive and active behaviors of female Wistar rats with high or low immobility time in the forced swimming test. Pharmacol. Biochem. Behav..

[43] Kusmider M., Solich J., Palach P., Dziedzicka-Wasylewska M. (2007). Effect of citalopram in the modified forced swim test in rats. Pharmacol. Rep..

[44] Brosen K., Naranjo C.A. (2001). Review of pharmacokinetic and pharmacodynamic interaction studies with citalopram. Eur. Neuropsychopharm..

[45] Duda-Chodak A., Tarko T., Satora P., Sroka P. (2015). Interaction of dietary compounds, especially polyphenols, with the intestinal microbiota: A review. Eur. J. Nutr..

[46] Garcia-Munoz C., Vaillant F. (2014). Metabolic Fate of Ellagitannins: Implications for Health, and Research Perspectives for Innovative Functional Foods. Crit. Rev. Food Sci..

[47] Basheer L., Kerem Z. (2015). Interactions between CYP3A4 and Dietary Polyphenols. Oxid. Med. Cell. Longev..

[48] Ahn D., Putt D., Kresty L., Stoner G.D., Fromm D., Hollenberg P.F. (1996). The effects of dietary ellagic acid on rat hepatic and esophageal mucosal cytochromes P450 and phase II enzymes. Carcinogenesis.

[49] Recamier-Carballo S., Estrada-Camarena E., Reyes R., Fernandez-Guasti A. (2012). Synergistic effect of estradiol and fluoxetine in young adult and middle-aged female rats in two models of experimental depression. Behav. Brain Res..

[50] Tam L.W., Parry B.L. (2003). Does estrogen enhance the antidepressant effects of fluoxetine?. J. Affect. Disord..

[51] Soares C.N., Poitras J.R., Prouty J., Alexander A.B., Shifren J.L., Cohen L.S. (2003). Efficacy of citalopram as a monotherapy or as an adjunctive treatment to estrogen therapy for perimenopausal and postmenopausal women with depression and vasomotor symptoms. J. Clin. Psychiat..

[52] Contreras C.M., Rodríguez-Landa J.F., Gutiérrez-García A.G. (2001). The lowest effective dose of fluoxetine in the forced swim test significantly affects the firing rate of lateral septal nucleus neurons in the rat. J. Psychopharmacol..

[53] Mostalac-Preciado C.R., de Gortari P., López-Rubalcava C. (2011). Antidepressant-like effects of mineralocorticoid but not glucocorticoid antagonists in th elateral septum: Interactions with the serotonergic system. Behav. Brain Res..

[54] Vega-Rivera N.M., Fernández-Guasti A., Ramírez-Rodríguez G., Estrada-Camarena E. (2013). Acute stress further decreases the effect of ovariectomy on immobility behavior and hippocampal cell survival in rats. Psychoneuroendocrinology.

[55] Detke M.J., Rickels M., Lucki I. (1995). Active Behaviors in the Rat Forced Swimming Test Differentially Produced by Serotonergic and Noradrenergic Antidepressants. Psychopharmacology.

